# Recovery Methods in Basketball: A Systematic Review

**DOI:** 10.3390/sports11110230

**Published:** 2023-11-20

**Authors:** Mladen Mihajlovic, Dimitrije Cabarkapa, Damjana V. Cabarkapa, Nicolas M. Philipp, Andrew C. Fry

**Affiliations:** Jayhawk Athletic Performance Laboratory—Wu Tsai Human Performance Alliance, Department of Health, Sport and Exercise Sciences, University of Kansas, Lawrence, KS 66045, USA

**Keywords:** nutrition, hydration, supplementation, sleep, tapering, cold-water immersion, rest, collegiate, professional, sport, athletes

## Abstract

Although different strategies have been implemented to manage recovery-fatigue status in athletes, there is still a lack of consensus on which recovery protocols have the greatest impact and effectiveness when implemented with basketball players, including both physiological and psychological recovery methods. Thus, the purpose of this systematic review is to: (a) determine which recovery methods attain the greatest benefit in restoring the process of attenuating fatigue and (b) provide sports practitioners with guidelines on how some of the most effective recovery strategies can be used to optimize athletes’ recovery and ultimately enhance their performance. Using the PRISMA guidelines, a total of 3931 research reports were obtained through four database searches (i.e., PubMed, Scopus, Cochrane, and Web of Science), from which only 25 met the inclusion and exclusion criteria. The recovery protocols analyzed in this systematic review were: sleep, nutrition, hydration, ergogenic aids, cold-water immersion, compression garments, massage, acupuncture, tapering, mindfulness, and red-light irradiation. The results revealed that all recovery strategies are capable of attenuating fatigue and enhancing recovery in basketball players to a certain degree. However, an individualized approach should be promoted, where a combination of proactive recovery modalities appears to result in the most rapid rates of recovery and athletes’ ability to maintain high-level performance. Recovery should be programmed as an integral component of training regimens. Also, cooperation and communication between coaches, players, and the rest of the team staff members are essential in minimizing the risk of non-functional overreaching or injury and optimizing basketball players’ on-court performance.

## 1. Introduction

Basketball is one of the most popular international sports. It is a fast-paced game characterized by repetitive movements of various intensity and duration and rapid changes in direction in both horizontal and vertical planes of motion [1,2,3]. Such movements include but are not limited to walking, pivoting, bilateral jumping, unilateral landings, and running with the change in the body orientation in relation to the direction of the movement [4]. Previous research has found that during a regular basketball game, players cover an average of 4500–5000 m [5,6], from which 56.8% is spent walking, 34.1% running and jumping, and 9.1% being stationary [4]. In addition, Abdelkrim et al. [7] have found that basketball players tend to change either their movement intensity or form every 2–3 s. Combined, this accumulates to 997 ± 183 movement changes throughout a 48-min game [8].

Although it is challenging to specify the exact contribution of each energy source due to different playing styles, it is well-accepted that basketball players primarily rely on the anaerobic metabolism to satisfy on-court competitive demands [1,3,7,9]. Possessing an adequate level of anaerobic power is of critical importance to satisfy the need for rapid force production over a short period of time, when performing repetitive acceleration, deceleration, change in direction, and jumping movements [9,10,11,12]. On the other hand, aerobic capacity is needed to sustain repeated short bouts of high-intensity exercise during the entire game, including both offensive and defensive actions [9]. As indicated by Meckel et al. [3], the contribution of the aerobic energy system is directly related to the amount of work performed by the basketball player during intermittent activity. Although being largely dependent on playing position, competitive level, and on-court competitive demands, maximal oxygen consumption (VO_2max_) in basketball players typically ranges between 41.9 and 74.4 mL·kg^−1^·min^−1^, with greater values being observed in guards when compared to forwards and centers [13,14,15].

Alongside the physiological demands during gameplay, the amount of off-court stress placed on the athletes needs to be considered. A recently published article by Holmes [16] revealed that National Basketball Association (NBA) teams that are likely to be playoff contenders flew on average 45,027 miles. For example, one of the teams accumulated 54,004 miles during a single competitive season, which is equivalent to flying from Chicago to Cleveland consecutively for 176 days [16]. This off-court stress has been found to have a negative impact on players’ hydration status, nutritional habits, quality and quantity of sleep, as well as overall well-being [17,18]. When combined with high physiological and psychological demands, prolonged recovery times between basketball training sessions and/or games are needed. In certain instances, inadequate levels of work-recovery balance may increase the likelihood of injury and illness [17,19]. Thus, considering the frequency of air travel in high-level competitive basketball, coaches, sports scientists, and strength and conditioning practitioners need to dedicate a considerable amount of attention to optimizing recovery and minimizing the negative impact of travel-related disturbances on athletes’ performance.

Rather than being observed in a hierarchical order, performance, recovery, and fatigue should be seen as a continuum affected by various physiological and psychological determinants [20]. While the exact definition is dependent on the condition under which it occurred, fatigue in a sport-specific setting can be defined as a feeling of exhaustion sustained by the inability to maintain the required level of performance [21]. Some of the mechanisms responsible for exercise-induced fatigue can be substrate depletion (e.g., glycogen, creatine phosphate), metabolites accumulation (e.g., lactate, hydrogen ions), hydro-electrolyte alterations (e.g., H_2_O, Na, K), and temperature elevation [22]. Usually, a decrement in physical performance occurs following an intense training session [23]. If there are appropriate periods of recovery time provided between the successive training sessions, a “supercompensation” may occur, where the athlete will demonstrate enhanced performance when compared to the baseline levels [23]. Conversely, if the training loads are too high (i.e., intensity and/or volume) or recovery time is inadequate, the effects of successive training sessions could result in a cumulative loss of physical capacity, which could further lead to overreaching or in extreme cases overtraining syndrome [23,24,25,26].

Overreaching is an accumulation of training and/or non-training stress resulting in short-term decrement in performance capacity with or without related physiological and psychological signs and symptoms of maladaptation in which restoration of performance capacity may take from several days to several weeks [25,27,28]. There are two levels of overreaching states—functional and non-functional [29]. Functional overreaching or short-term overreaching is a consequence of increased training load that leads to a temporary performance decrement and improvement in performance after rest—supercompensation [25,29]. On the other hand, non-functional overreaching is characterized by notably greater performance decrements accompanied by neuroendocrine and/or psychological symptoms, and it requires a considerably greater amount of rest for performance levels to be completely restored [25,29]. If training loads are not modified, overreaching can progress into overtraining that induces alterations in several physiological systems, including long-term performance decrement despite continued training [24]. The main difference between overreaching and overtraining is in the time needed for the complete restoration of performance capacity which can last weeks or sometimes even months, with or without related physiological signs or symptoms [23,24,30]. Some of the parasympathetic alterations common for aerobic sports include fatigue, depression, bradycardia, and loss of motivation, while for anaerobic sports such as basketball, common symptoms have shown to be irritability, agitation, tachycardia, hypertension, and restlessness [30]. Other symptoms that may occur depending on the specificity of the training activity that leads to the overtraining state include but are not limited to heart rate variability alterations, muscle soreness, weight loss, lack of mental concentration, anxiety, and changes in an enzyme (e.g., creatine kinase) and hormonal concentrations (e.g., testosterone, cortisol) [24,25,30].

Many fatigue monitoring tools are currently available to coaches, sports scientists, and strength and conditioning practitioners to monitor performance capabilities and perceived fatiguability levels in basketball players [31]. Some of the key performance indicators are sprinting and vertical jump ability, athlete self-report measures, heart rate indices, and biochemical markers [8,31,32,33,34,35]. The countermovement vertical jump is a commonly used testing modality to assess neuromuscular performance and a level of fatigue in sport-specific settings [36,37]. When performed on innovative force plate systems that allow for rapid data analysis, a plethora of performance metrics can be obtained, focused on examining both eccentric and concentric phases of the movement [11,12,36,37,38,39]. Besides solely being focused on examining jump height as an outcome metric, a detailed examination of force-time variables (e.g., peak concentric force, breaking phase duration, countermovement depth) may help practitioners monitor and quantify recovery-fatigue neuromuscular changes in basketball players to a greater extent [36,37,40]. In addition, physiological and psychological measures of fatigue represent an important component of the recovery-fatigue monitoring process in athletes [20]. Some of the physiological markers of disruption, stress, and inflammation include creatine kinase, blood lactate, cortisol, testosterone, and insulin-like growth factor [20]. For example, creatine kinase has been found to be a reliable blood-related marker of recovery-fatigue status in team sport athletes [41,42]. Additionally, a recently published study focused on examining salivary hormonal changes during a basketball game found a significant increase in cortisol post-third-quarter and a decrease in the testosterone-to-cortisol ratio 30 and 60 min post-game when compared to the baseline levels [32]. However, it should be noted that in certain instances creatine kinase and cortisol may need >72 h to fully recover, despite physical performance returning to the baseline levels 48 h post-competition, indicating the need for additional recovery time [43].

Alongside several physiological performance assessments, psychological measurements of acute and/or chronic responses to training or competitive loads commonly used in sport-specific settings have been the rating of perceived exertion (RPE), Profile of Mood States, and Recovery-Stress Questionnaire for Athletes [20]. A recently published study focused on examining a cohort of professional male volleyball players found a strong positive relationship between the RPE and algorithm-derived external load as well as the number of jumps completed during a regular in-season training session [38]. Similar findings apply to the game of basketball, where RPE is demonstrated as a viable tool for the assessment of internal loads imposed by training and competition on various levels of play [44,45]. In addition, Lochbaum et al. [46] have found that the Profile of Mood States scales were a solid predictor of sports performance in a variety of sports, while Nunes et al. [47] have found that the Recovery-Stress Questionnaire for Athletes adequately resembled changes in the periodized training plan load in female basketball players targeted toward optimizing on-court competitive performance.

Considering the aforementioned physiological and psychological methods of fatigue assessment in athletes, it is important to mention some of the commonly used techniques to speed up the recovery process. Based on Kellmann et al. [20], the recovery process can be subdivided into passive (e.g., massage or total inactivity), active (e.g., low-intensity physical activity), and proactive (e.g., social activities with a high level of self-determination). Which recovery method will be implemented primarily depends on the type of activity performed, practice and competitive schedules, and the availability of the team staff to complete the specific activity [48]. In addition, it should be noted that post-exercise recovery strategies can also be classified as “primary” (e.g., sleep, nutrition, hydration) and “secondary” (e.g., ergogenic aids, cooldown strategies, manual therapy) [49]. The primary are the ones that need to be prioritized, and if needed, they can be further supplemented with secondary recovery strategies to maximize the effectiveness and efficiency of the overall recovery process and prepare athletes for the upcoming on-court competitive demands [49].

Based on the comprehensive review of the scientific literature, it is obvious that a variety of tools have been used in sport-specific settings to monitor recovery-fatigue status in athletes. However, there is still a lack of consensus on which recovery strategies have the greatest impact and effectiveness when implemented with basketball players, including both physiological and psychological recovery methods. Thus, the purpose of this systematic review is to: (a) determine which recovery methods attain the greatest benefit in restoring the process of attenuating fatigue and (b) provide coaches, sports scientists, and strength and conditioning practitioners with guidelines on how some of the most effective recovery strategies can be used to optimize athletes’ recovery and ultimately enhance their on-court basketball performance.

## 2. Materials and Methods

### 2.1. Data Sources

The systematic review was conducted based on Preferred Reporting Items for Systematic Reviews and Meta-Analysis (PRISMA) recommendations [50]. The literature search process for relevant research articles published before 15 August 2023 included PubMed, Scopus, Cochrane, and Web of Science databases. The following keywords were introduced, and used in combination with the Boolean operators: recovery OR stretching OR static stretching OR active recovery OR passive recovery OR foam roll OR massage OR vibration therapy OR whole body vibration OR electrostimulation therapy OR electrostimulation OR cool down OR proprioceptive neuromuscular facilitation OR sauna OR jacuzzi OR cold water immersion OR contrast water therapy OR contrast bath OR ice bath OR cold shower OR cryotherapy OR ice massage OR ice therapy OR cold pack OR compression garment OR hydration OR isotonic drink OR carbohydrate OR protein OR diet OR rest OR sleep OR branched-chain amino acid OR glutamine OR L-glutamine OR creatine OR creatine monohydrate OR energy gels OR beta-alanine OR compression pants AND basketball. In addition, the reference lists were screened to check if other relevant studies fit the inclusion criteria. The PRISMA checklist for this systematic review provided in the supplementary materials.

### 2.2. Inclusion and Exclusion Criteria

The studies included in this systematic review were selected based on the following criteria: (a) participants completed at least one recovery session consisting of any type of recovery method (e.g., nutritional, psychological); (b) participants were all basketball players competing at professional (e.g., national team, international leagues) or amateur level (e.g., collegiate, recreational); (c) countermovement vertical jump, sprint-time, muscle power and force, VO_2max_, blood lactate concentration, creatine kinase, RPE, and muscle soreness were outcome variables regarding the recovery process. The studies excluded from this systematic review were the ones that did not meet the following criteria: (a) manuscripts not published in English; (b) manuscripts with no fully available text; (c) no recovery-related intervention protocol being administered; (d) participants were not only basketball players; (e) participants were younger than 17 years old; (f) participants were wheelchair basketball players; (g) a cohort of participants included injured players.

### 2.3. Methodological Quality

The initial keyword database search and methodological quality was performed by the lead author. Upon the completion of the search process, all the articles retrieved from the selected databases were uploaded into a reference manager software (Zotero, Corporation for Digital Scholarship, Vienna, VA, USA). Then, all the articles were screened for duplicates and the titles and abstracts were read to exclude those that were not related to the topic of this systematic review. The remaining research reports included in this systematic review were screened for their methodological quality via the Physiotherapy Evidence Database (PEDro) scale [51].

## 3. Results

**Figure 1:**
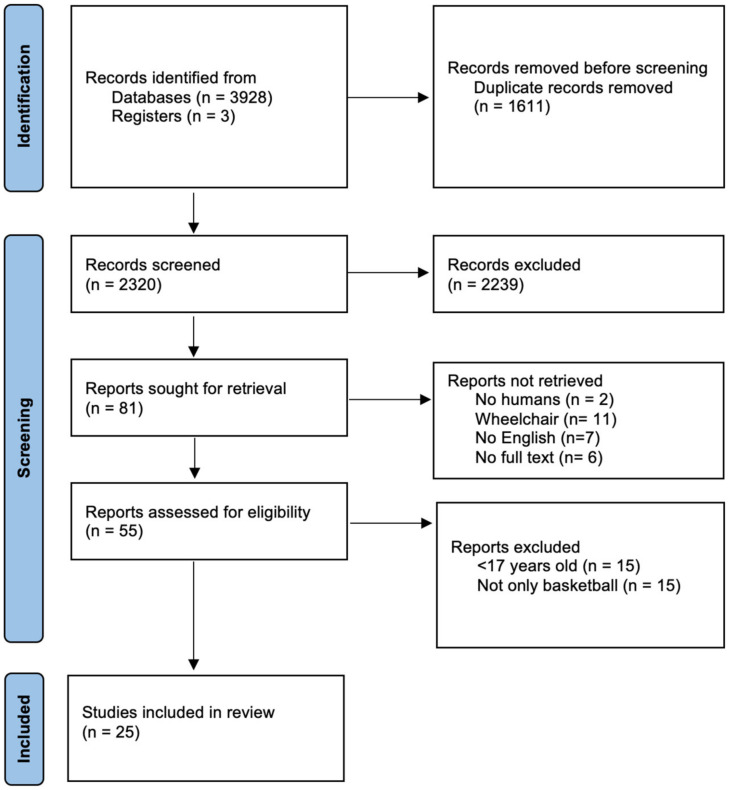
Flow diagram of the scientific literature selection process.

The initial search yielded 3928 articles from four different databases and three articles from other sources. After the duplicates were removed, 2320 remaining articles were screened by titles and abstracts. Then, 81 articles with full text available were selected to be reviewed and assessed by inclusion and exclusion criteria. Following the exclusion of the unqualified research reports, a total of 25 articles were included in this systematic review (Figure 1).

From the studies included in this systematic review, a total of 444 basketball athletes were analyzed and all played on amateur (*n* = 355/79%) or professional (*n* = 89/21%) levels of basketball competition. The overall sample of participants was composed of 352 male and 67 female athletes. The sample size ranged between 8 and 30. The ages of participants ranged between 17 and 32. Most of the studies were gender-specific, while two studies included a mixed sample [52,53] and two were gender-unspecified [54,55].

One of the main differences observed among qualified research reports is the type of recovery protocol implemented with the athletes, which aligns with the main objective of this systematic review aimed to compare and understand which recovery strategies yield better adaptations and attain the greatest benefit in restoring the process of attenuating fatigue. The recovery protocols included in the systematic review were based on nutrition/supplementation, psychology (e.g., mindfulness), tapering, physio-prophylactic procedures (e.g., cold water immersion, massage, stretching), rest/sleep, hydration, or other (e.g., compression garments, acupuncture, and red-light therapy). On the other hand, some of the most common dependent variables used to quantify the recovery-fatigue process were jump height, cortisol, testosterone, creatine kinase, hemoglobin, myoglobin, alanine transaminase, aspartate aminotransferase, maximal isometric voluntary contraction, RPE, total quality recovery, and muscle soreness assessment. In addition, for physical performance, recovery-fatigue assessment modalities included a 20 m sprint, repeated sprint test (6 × 20 m), 5-0-5, 300-yard shuttle run, reaction time, and sit-and-reach test. The detailed description of recovery methods and testing procedures examined in this systematic review as well as their effectiveness are presented in Table S1 (Supplementary Materials).

## 4. Discussion

The main objective of this systematic review was to examine the effects of different recovery methods on physiological and psychological performance parameters in basketball players and to determine which of the selected recovery protocols can induce the greatest benefit in restoring processes or attenuating fatigue. Despite the variety of intervention protocols and their duration, almost every study included in this systematic review revealed significant improvements in the outcome variables. These results can provide coaches, sports scientists, and strength and conditioning practitioners working with basketball players with beneficial information that can be used to select adequate recovery methods as a part of the short-, mid-, and long-term periodization planning process.

### 4.1. Sleep

Sleep is one of the key factors that have a direct or indirect effect on sports performance [56,57]. When considering overall physiological demands, athletes need 7–9 h of sleep, where 80–90% needs to be accumulated throughout the night [56,58]. Mah et al. [59] investigated the effect of sleep extension as a recovery method over a 5–7-week period on athletic performance in collegiate basketball players. The results revealed that sleep extension (i.e., >10 h in bed) was positively associated with faster spirit times and free-throw and three-point shooting performance (i.e., ~9% increase in shooting accuracy) [59]. Moreover, the athletes reported lower Psychomotor Vigilance Task and Epworth Sleepiness Scale scores and higher ratings of overall physical and mental well-being following the sleep extension [59]. Similar findings emerge from an investigation conducted by Jones et al. [60] focused on examining the association between the late-night presence of social media and next-day performance among professional male basketball players. It has been found that a greater amount of time spent on social media is negatively associated with the number of rebounds and points scored during a game and positively associated with the number of turnovers and personal fouls committed [60]. In addition, previous research has found an acute increase in heart rate, ventilation, and ventilation to VO_2max_ ratio at submaximal and maximal workloads following a night of sleep deprivation, implying that sleep loss can negatively impact endurance performance [61]. Although focused on examining a cohort of collegiate soccer players, Ajjimaporn et al. [62] found that three hours of sleep per night had a negative effect on anaerobic performance, muscle strength, and fatigue during the next-day afternoon testing session. Also, it should be noted that increased sleep duration (i.e., >8 h) was associated with lower injury rates as well as higher feelings of subjective well-being in collegiate male basketball players [63]. Interestingly, similar findings pertaining to a decrease in the likelihood of injury with an increase in sleep duration were observed by a few other research reports when examining NCAA Division-I female volleyball players and elite male soccer players [42,64,65]. Thus, according to the aforementioned research reports, we can conclude that not just sleep but sleep extension is of critical importance to athletes, including basketball players, as it can have a positive impact on improvements in physical performance (e.g., sprinting, reaction time, shooting accuracy) as well as decrease likelihood of injury occurrence.

### 4.2. Nutrition

A well-structured and planned nutrition regimen is another crucial factor that needs to be considered to optimize recovery in athletes [17]. Adequate nutrition helps sustain and enhance performance demands. Congested practice and game schedules, including travel demands, may diminish athletes’ ability to consume an adequate amount of nutrients, which may ultimately jeopardize the recovery process and on-court basketball performance [17,49]. In a recently published study, Ho et al. [54] investigated the effects of ingestion of high-protein content (i.e., 36% of total calorie intake) on the recovery-fatigue process in collegiate basketball players. When compared to the low-protein test trial (i.e., 12% of total calorie intake), high-protein supplementation enhanced cerebral oxygen saturation during the cycling performance test and attenuated increases in cerebral blood perfusion alongside improving total cycling time by 16% [54]. In a similar investigation, Ronghui [55] investigated the effect of ingestion of whey protein powder (i.e., 20 g) and oligosaccharides (i.e., 40 g) dissolved in 250 mL of whole milk on the recovery process in amateur basketball players following a one-month long training session on a cycle ergometer. The authors found that the supplementation group attained significantly higher hemoglobin, hematocrit, and red blood cell count than the control group that consumed only 250 mL of milk post-exercise [55]. Combined, these findings suggest that the consumption of a high-protein diet as a recovery method has the potential to enhance exercise capacity and diminish the impact of fatigue on athletes’ physical performance capabilities. Additionally, it is important to note that even if carbohydrate is the primary energy source needed to replace adenosine triphosphate and creatine phosphate during high-intensity exercise [7,66], consumption of carbohydrate and protein mixture has been found to positively impact the recovery process and improve subsequent exercise performance [67]. Based on the findings of Beelen et al. [67], ingestion of a small amount of protein (i.e., 0.2–0.4 g·kg^−1^·h^−1^) with carbohydrates (i.e., 0.8 g·kg^−1^·h^−1^) resulted in similar muscle glycogen-repletion rates as the ingestion of high dose of carbohydrates (i.e., 1.2 g·kg^−1^·h^−1^). Although further research is warranted to examine mechanisms of how protein supplementation increases oxygen saturation during exercise, the aforementioned findings solidify the importance of adequate nutrition to maintain peak performance demands and optimize recovery in high-intensity sports such as basketball.

### 4.3. Hydration

Starting exercise in a well-hydrated state (e.g., pre-exercise hydration) and consumption of appropriate amounts of fluids during exercise is essential for peak athletic performance [68]. A study conducted by Howard & Hillman [69] investigated the influence of hydration on cognition and skill performance in female basketball players. The authors found that the decrease in plasma volume was notably lower when the athletes consumed a carbohydrate-electrolyte drink when compared to athletes who consumed just water [69]. Also, the same group of athletes demonstrated better memory test results alongside having lower blood lactate concentrations, suggesting that not just the amount but the type of fluid also needs to be considered when attempting to maintain adequate hydration levels during exercise [68,69]. These findings are further supported by Carvalho et al. [70] who found that weight loss (i.e., dehydration) during exercise was significantly lower when the athletes consumed a carbohydrate-electrolyte drink rather than just water, and was lower when consuming water over no fluid intake at all. The athletes consuming carbohydrate-electrolyte solution during exercises reported the lowest RPE scores, while no differences in basketball-specific drills (e.g., two-point shooting, sprinting) between the conditions were observed [70].

A considerable loss of water and inadequate electrolyte balance can impair both physiological and psychological performance and eventually diminish athletes’ ability to compete [68]. A 1–2% dehydration may impair athletes’ concentration, vertical jump performance, aerobic endurance, weaken cognitive function, reduce efficiency, and lower exercise tolerance [71,72,73]. Although focused on examining a cohort of nationally ranked male and female tennis players, McRae & Galloway [74] found that ingestion of a carbohydrate-electrolyte drink elevated blood glucose concentration throughout the match as well as athlete’s energy-level feeling when compared to the placebo condition. Also, athletes who consumed a carbohydrate-electrolyte drink spent more time in moderate-intensity than low-intensity activity than in the placebo condition [74]. In addition, it should be noted that previous research has found a positive relationship between L-alanyl-L-glutamine intake and hydration status when examining NCAA Division-I collegiate female basketball players [75]. Rehydration with L-alanyl-L-glutamine appeared to be beneficial in maintaining visual reaction time and basketball skill performance [75]. Specifically, jump-shooting accuracy was 12.5% and 11.1% greater when low-dose L-alanyl-L-glutamine (i.e., 1 g per 500 mL) was mixed with water when compared to both no-fluid or just water consumption, respectively [75]. This study further confirms previously mentioned findings pertaining to the importance of fluid and electrolyte intake during exercise to prevent dehydration and ultimately impair basketball performance.

### 4.4. Supplements

Alongside its impact on hydration, the effect of L-glutamine has also been studied as a recovery-enhancing supplement. Cordova-Martinez et al. [76] used a double-blind crossover study design to investigate the impact of L-glutamine supplementation on muscular disruption biomarkers in professional male basketball players during a competitive season span. Significant decreases in aminotransferase, creatine kinase, myoglobin, and adrenocorticotropic hormone were observed following L-glutamine ingestion (i.e., 6 g per day for 20 days) when compared to the placebo group [76]. Also, cortisol as a marker of the catabolic state remained unaltered between the testing time points in the experimental group [76]. This can be of great benefit to promote recovery, considering that creatine kinase and lactate dehydrogenase were 210% and 110% greater the morning after the basketball game when compared to the baseline levels [77]. These findings suggest that L-glutamine supplementation can attenuate exercise-induced muscle disruption in an intermittent team sport such as basketball where players are required to repetitively perform high-intensity acceleration, decelerations, jumping, and change-of-direction movements. In addition, another supplement that has been found to be beneficial for the delay in neuromuscular fatigue during a simulated basketball game is sodium bicarbonate (NaHCO_3_) [78]. Although no differences in 15 m sprint time and layup completion efficiency were observed, a significantly lower reduction in maximal voluntary isometric contractions (i.e., 100 Hz and 10 Hz) was noted in the experimental group, suggesting that sodium bicarbonate supplementation may protect contractile properties of the muscle during high-intensity exercise stimulus [78].

The effect of branched-chain amino acids (BCAA) and arginine (i.e., 0.17 g·kg^−1^ and 0.04 g·kg^−1^) supplementation on the physical and skill performance of collegiate basketball players was examined by Lin et al. [79]. The authors found no differences in shooting percentage and plasma markers for carbohydrate and fat metabolism between the experimental and placebo testing trial, but the time needed to complete vertical jumps, suicide sprints, and key and full court combinations drills was notably lower when the athletes received BCAA and arginine supplementation [79]. Also, it should be noted that higher post-exercise plasma BCAA concentration was found during the supplementation trial, as well as lower tryptophan/BCAA ratio [79]. Similar findings were observed by Chen et al. [80] when studying the recovery-fatigue process within a cohort of taekwondo athletes across three simulated matches. Although additional research is warranted, the observed reduction in the tryptophan/BCAA ratio could be one of the potential mechanisms responsible for the alleviation of central fatigue [79,80]. Furthermore, Hassmen et al. [81] reported that supplementation with BCAA could prevent an increase in the tryptophan/BCAA ratio in cross-country athletes as well as improve performance on various complex types of cognitive tests. Additionally, while BCAA supplementation can have a positive impact on delaying fatigue, it is important to take into account the ratio between leucine, isoleucine, and valine. A recently published investigation found that a leucine-enriched amino acid mixture can suppress the elevation of muscle disruption blood markers (e.g., creatine phosphokinase) post-exercise in untrained males [82]. Therefore, based on the previously mentioned research reports, it can be concluded that supplementation with BCAA, especially leucine, may be an effective strategy to help optimize post-exercise recovery in basketball players.

Other supplements that have been found to benefit the recovery process in basketball players by decreasing oxidative stress levels are Vitamin E, Vitamin C, and β-carotene [83]. Daily ingestion of 600 mg of Vitamin E, 1 g of Vitamin C, and 32 mg of β-carotene over a period of 32 days during a competitive season induced a significant decrease in lipoperoxides, including a 15.3% decrease in the lipoperoxides/total antioxidant status ratio as an indicator of lower oxidative stress [83]. In addition, in the same investigation, the authors detected a significant decrease in Vitamin C concentrations pre-post testing timeline in the placebo group [83]. These findings are further supported by Naziroglu et al. [84] who found that supplementation with Vitamin C and Vitamin E may strengthen the antioxidant defense system in basketball players during high-intensity training loads.

### 4.5. Cold-Water Immersion

Based on the scientific literature included in this systematic review, a couple of studies have investigated the effects of cold-water immersion (CWI) on physical performance parameters, muscle soreness, RPE, blood-related markers of muscle disruption, and blood lactate concentration in basketball players [52,85,86,87,88]. Each of them implemented a slightly different CWI protocol, with the main difference being in the water temperature. For example, Chaiyakul & Chaibal [85] found significantly lower perceived muscle soreness levels 24 h post-high-intensity intermittent exercise in the CWI group (i.e., 15 min at 15 °C) when compared to the control group (i.e., no CWI). Also, while no changes pre-post exercise were observed within the CWI group, vertical jump height was notably lower than pre-testing values [85]. The positive impact of CWI (i.e., 5 × 1 min at 11 °C) on a decrease in muscle soreness levels was also observed by Montgomery et al. [86], while Delextrat et al. [52] found that similar CWI treatment (i.e., 2 min at 11 °C with 2 min break at 20 °C) resulted in the improvement of vertical jump performance 24 h post-game. In addition, Seco-Calvo et al. [88] have observed the benefits of intermittent CWI recovery protocol on blood-related markers of muscle disruption such as lactate dehydrogenase, creatine kinase, and aldolase. Analogous findings pertaining to a decrease in blood lactate concentration following continuous CWI treatment (i.e., 16 °C for 7 min) have been observed by Pelana [87]. Therefore, based on the aforementioned research reports, we can conclude that both intermittent and continuous CWI treatment modalities can be effective in minimizing stress responses and optimizing recovery in basketball players. Additionally, CWI was found to be a more effective intervention than static stretching or passive recovery [89,90,91]. However, considering that a study conducted by Peake et al. [92] offers contradictory findings indicating that CWI is no more effective in reducing muscle inflammation and cellular stress following a bout of resistance exercise, further research is warranted on this topic.

### 4.6. Compression Garments

Throughout the literature review process, three studies that used compression garments as a recovery method in basketball players emerged. More specifically, Atkins et al. [93] and Ballmann et al. [94] focused on examining the effectiveness of using compression garments as a recovery treatment modality, while Fernández-Lázaro et al. [95] studied compressive cryotherapy as a non-pharmacological muscle recovery strategy. Atkins et al. [93] found non-significant differences in all performance measures (i.e., sprinting, jumping, and agility) between the group of recreationally active male basketball players that used compression garments and the control group following fatigue-inducing training sessions in the duration of approximately two hours. However, the same group of authors found that perceived post-recovery fatigue and muscle soreness were significantly lower for the experimental when compared to the control group, while the sleep quality was considerably enhanced [93]. Interestingly, when the same recovery method was implemented with male rugby players, no performance and recovery improvements were observed [96]. The only benefit of using compression garments was decreased muscle soreness, which agrees with the aforementioned findings [93,96]. On the other hand, a study focused on examining NCAA Division-I male basketball players found that wearing compression garments increased mean power output during Wingate Anaerobic Test (i.e., 2 × 30 s) anaerobic capacity and total work performed [94]. Moreover, RPE reported while wearing compression garments was significantly lower when compared to the control group [94]. In addition, to the best of the authors’ knowledge, compression cryotherapy (i.e., 8 °C, 2 × 15 min) was examined for the first time in the study by Fernández-Lazáro et al. [95]. A progressive decrease in multiple muscle disruption markers (e.g., creatine kinase, aspartate transaminase, alanine transaminase, and myoglobin) was observed in the experimental group (i.e., compression cryotherapy) at the end of the pre-season competitive period when compared to the control group [95]. Moreover, a significant increase in creatine kinase was observed in the control group, while the experimental group experienced a notable decrease in RPE [95]. According to the results of the previously mentioned studies, the usage of compression garments or compressive cryotherapy was shown as an effective recovery method when implemented with basketball players.

### 4.7. Massage

While there are different types of massage, a few studies have focused on examining its effect as a recovery method in basketball players [52,53,97]. Delextrat et al. [53] investigated the effect of massage and massage combined with stretching on post-game recovery in national-level male and female basketball players. In male athletes, both treatment modalities demonstrated beneficial effects on vertical jump performance, while in female athletes repeated sprint ability had a smaller decrement after combined treatment (i.e., massage and stretching) following a basketball match [53]. Moreover, both recovery protocols improved perceptions of fatigue and soreness [53]. These findings are further supported by the follow-up investigation conducted by the same group of authors [52]. Similar findings were observed by Kaesaman & Eungpinichpong [97] who studied the effect of Traditional Thai Massage on recovery following basketball-specific exercise. Only 10 min recovery protocol was capable of inducing a significant increase in heart rate variability post-exercise [97]. Interestingly, while a recently published systematic review found no direct impact on sports performance, including massage as a part of recovery protocols may improve flexibility (i.e., range of motion) and delayed onset muscle soreness [98]. Also, the mechanical pressure on the muscle is expected to induce changes in parasympathetic activity (e.g., heart rate, blood pressure, heart rate variability) and hormonal levels (e.g., cortisol) that will ultimately cause a relaxation response [99]. Moreover, including massage as a part of regular recovery regimens may reduce an athlete’s anxiety and improve their mood state which can additionally optimize recovery [99]. Thus, despite the questionable impact on physical performance directly, sports massage and its variations could be one of the methods used to optimize recovery in basketball players. These findings can help sports practitioners better understand the effects of massage and how it can be used to manage various conditions caused by exercise-induced fatigue.

### 4.8. Acupuncture

Acupuncture is a traditional Chinese medicine technique that has been used for thousands of years to treat illness or pain management. Also, it has been used to improve exercise-induced muscle fatigue. Lin et al. [100] examined the effect of acupuncture stimulation on recovery on physiological performance parameters in male collegiate basketball players. The findings reveal that athletes who underwent acupuncture treatment (i.e., PC6 and ST36 acupoints) demonstrated significantly lower maximal heart rate, VO_2max_, and blood lactate values 30 min post-exercise when compared to the control group (i.e., no acupuncture treatment) [100]. Moreover, the blood lactate concentrations remained notably lower for the experimental group 60 min post-exercise [100]. Similar findings pertaining to the benefit of acupuncture treatment for improvements in recovery-fatigue status (i.e., decrease in blood lactate, pyruvate, citrate) after a series of exhaustive bouts of high-intensity exercise with short rest periods were noted by Ma et al. [101] when examining a cohort of moderately trained males. Additionally, a couple of other research reports investigated the effect of acupuncture in recreationally active individuals [102,103]. Lin & Yang [102] found a positive effect of acupuncture treatment on exercise-induced muscle soreness but not on serum creatine kinase activity. The perception of muscle soreness was lower 72 h following the exercise session in the group that received the acupuncture treatment when compared to the control group, which was also confirmed in the findings of Hubscher et al. [103]. Although further research is warranted on this topic, the aforementioned results indicate that basketball players may benefit by incorporating acupuncture treatment in their recovery protocols through a decrease in post-exercise heart rate, oxygen consumption, and blood lactate, as well as the perception of muscle soreness and fatigue.

### 4.9. Tapering

The taper represents a progressive reduction in training load over a specific period with the purpose of reducing both physiological and psychological stress induced by training and competitive demands to optimize athletes’ performance capabilities [104]. Based on the currently available scientific literature, the properly implemented tapering strategies are capable of inducing on average 3% improvement in performance (i.e., 0.5–6.0%). Svilar et al. [105] have investigated the impact of a short-term reduction in training load on recovery status in top-level professional male basketball players. The authors found that a decrease in both internal and external training loads three days prior to the official game can have a positive impact on a player’s readiness to compete [105]. To the best of the authors’ knowledge, this was the first study that documented the positive impact of implementing short-term tapering strategies to optimize recovery in basketball players. On the other hand, a two-week tapering period in team sports such as soccer and handball prior to the competition further supports the positive impact of the reduction in training load on subsequent game performance as well as players’ recovery status [106,107]. For example, Beltran-Valls et al. [106] found a two-week tapering period (i.e., a 50% reduction in training volume) to be an effective strategy to increase muscle power and acceleration and reduce the perception of fatigue in amateur male soccer players. A similar tapering strategy was used by Hermassi et al. [107] with a cohort of elite male handball players, which resulted in an increase in muscle power, faster sprinting times, and ball-throwing velocity following a 10-week of progressive resistance training (i.e., 60% reduction in training intensity). Also, it should be noted that the findings of a recently published study conducted on 158 elite junior male soccer players indicate that four-week exponential tapering (i.e., training load decrease in a nonlinear manner) produced better performance outcomes than linear tapering (i.e., constant decrease in training load) following four-weeks of training [108]. The exponential tapering strategy produced better results in speed (i.e., 5 and 30 m sprint), power (i.e., countermovement vertical jump), and endurance abilities (i.e., VO_2max_) than the linear strategy [108]. All considered, implementing various types of tapering strategies by reducing the training volume may help with fatigue reduction and physical performance optimization prior to the competition. Considering the observed benefits of the previously mentioned recovery strategies (e.g., hydration, massage, supplementation), adequately implemented tapering methods can be of great benefit to basketball players.

### 4.10. Mindfulness

Mindfulness is a form of meditation that is emerging as a very popular psychological practice in sports in which an individual becomes intensively aware of their sensations and feelings [109]. It allows athletes to better cope with anxiety as well as improve overall cognitive function [110,111]. Vidic et al. [112] investigated the impact of ten mindfulness-based sessions on collegiate basketball players and found that the intervention elicited a progressive decrease in stress and an increase in athletic coping skills. Moreover, the athletes who practiced mindfulness in different aspects of life demonstrated an increase in awareness, control, focus, presence, and relaxation, suggesting that it can be beneficial in both athletic settings and everyday life [112]. To couple with these findings, Ajilchi et al. [113] found that sports mindfulness training was positively related to mental toughness and emotional intelligence in athletes. In addition, alongside confirming the previously mentioned findings, it has been found that an eight-week-long mindfulness training session with a collegiate female rowing team can elicit improvements in subjective and objective sleep quality and athletic performance measured by a 6000 m ergometer test [114]. In an investigation focused on examining amateur male baseball players, significant improvements in flow state were observed following a one-month mindfulness workshop [115]. Also, the individuals who completed mindfulness training reported improvements in depressive symptoms, attention, and working memory [116]. Lastly, previous research has found that athletes who reported greater mindfulness scores had a decreased likelihood of injury occurrence [117]. Therefore, we can conclude that mindfulness can be used as a successful tool to help athletes improve their on-court and off-court demands. Considering that basketball players experience a considerable amount of stress, both physiological and psychological, and that they are constantly exposed to condensed practice, competition, and travel schedules, this technique can be used to optimize athletes’ mental health which may ultimately improve their athletic performance. Also, it may be beneficial that sports organizations designate individuals with training in sport psychology/mindfulness to work with players on implementing mindfulness as a regular daily routine and create a positive caring team environment.

### 4.11. Red-Light Therapy

Last but not least, red-light irradiation, commonly referred to as red-light therapy, has occasionally shown beneficial when implemented in sport-specific settings. However, there is a limited amount of scientific literature currently available on this topic, and further research is warranted before firm conclusions pertaining to its effectiveness can be made. Zhao et al. [118] have found that exposure to a red-light irradiation every night for 30 min over a period of two weeks improved sleep quality in elite female basketball players and indirectly induced improvements in recovery and overall well-being. Also, in the same investigation, the authors found that red-light therapy significantly improved serum melatonin levels and endurance performance (i.e., Cooper 12-min run) [118]. Although there seems to be a benefit of using whole-body red-light irradiation in a recovery manner, it should be noted that the logistics may be an issue due to transport and installation, especially when taking into account constant and condensed travel schedules that some amateur or professional basketball teams may have.

### 4.12. Limitations

Despite offering a deeper insight into some of the most commonly used recovery methodologies in a basketball-specific setting, this systematic review is not without limitations. There is a high heterogeneity observed between the studies included in the systematic review regarding the participants’ characteristics (e.g., amateur or professional athletes), recovery methods (e.g., CWI, sleep extension, hydration, compression garments), and training protocols (e.g., simulated basketball game, three-day tournament), which limits direct comparison between the studies and generating concise conclusions and recommendations for sports practitioners working with basketball players on which recovery methods should be prioritized. Also, only research reports published in the English language including participants >17 years old were included in this review, which could have introduced a language and/or age bias. To the best of our knowledge, no research reports replicated the real demands of the basketball game during the regular competitive season where the weekly or monthly condensed training schedule is influenced by the time available until the next game and the level of stress that athletes were exposed to. Alongside addressing the aforementioned issues, future research needs to determine whether a single or combination of different recovery strategies could be more effective and applicable when working with amateur and professional basketball players.

## 5. Conclusions

Recovery should be programmed as an integral component of training regimens. Based on the research reports included in this systematic review we can conclude that all the discussed recovery strategies (e.g., sleep, nutrition, hydration, CWI, supplementation, compression garments, tapering) can attenuate fatigue and enhance recovery in basketball players to a certain degree. Sleep, nutrition, and hydration are the elementary needs of any human and they should be prioritized to maximize players’ recovery. If needed, they can be encompassed by secondary recovery strategies that together can optimize the recovery process and assure that the athlete is properly prepared for the upcoming competitive demands.

## 6. Practical Applications

Mindfulness should be considered one of the most adequate and non-invasive methods that can have a notable positive impact on athletes’ performance both on and off the court, as well as an indirect influence on sleep and overall well-being. Although all recovery methods showed improvements in delayed onset muscle soreness, it seems that soft tissue treatment such as massage and/or stretching can have additional benefits to enhance recovery of the locomotion system post-exercise. Furthermore, adequate hydration, nutrition, and supplementation are the only recovery methods that can promote and maintain adequate fluid and nutritional needs throughout the exercise.

Lastly, optimal recovery should consist of a positive perception of recovery itself, while addressing the appropriate physiological and psychological mechanisms that are necessary to recover from training sessions and competition. Considering the implementation of recovery strategies in basketball, an individualized approach should be promoted, where a combination of proactive recovery modalities appears to result in the most rapid rates of recovery and athletes’ ability to maintain high-level performance. Also, cooperation and communication between coaches, players, and the rest of the team staff members are essential in minimizing the risk of non-functional overreaching or injury and optimizing basketball players’ on-court performance.

## Data Availability

No new data are generated in the present manuscript.

## Supplementary Materials

The following information can be downloaded at: https://www.mdpi.com/article/10.3390/sports11110230/s1. The PRISMA checklist. Table S1. Overview of research studies included in the systematic review. Reference [119] are cited in the supplementary materials.
