# Supplementary material for: Recovery Methods in Basketball: A Systematic Review
Source: Sports (Basel). 2023 Nov 20;11(11):230. doi: 10.3390/sports11110230 (PMC10675622; doi:10.3390/sports11110230)
Supplement: Supplementary file 1 [file sports-11-00230-s001.zip › sports-2679388-supplementary.pdf]

Table S1. Overview of research studies included in the systematic review.

Study	Subjects	Exercise protocol	Measurement	Measurement timing	Recovery methods	Findings
Ansdell & Dekerle (2020)	10 amateur male basketball players	Basketball game simulation	MVIC (10 and 100 Hz), 15m sprint time, and layup completion	Before and after each quarter of a simulated basketball game	0.2 g/kg of $NaHCO_3$ 90 and 60 min pre-game	↑* MVIC were greater in the experimental group (10 and 100 Hz)<br>↔ between groups in 15m sprint times and layup completion (both decreased)
Atkins et al. (2020)	30 recreationally active male basketball players	2x12 min of Basketball Exercise Simulation Test, 4x10 lunge jumps, and 2 min wall sits	Muscle soreness, CMJ, RST 6x20m, 505 agility test, and sleep quality	Before and after the testing protocol and 15h following exercise	Lower body compression garments (15h after exercise - overnight)	↑* perceptual ratings of fatigue and muscle soreness<br>↔ or unclear effects for performance measures
Ballmann et al. (2019)	12 male NCAA Division-III basketball players	Wingate anaerobic test (2x30sec) with 5min recovery	Mean and peak power, anaerobic and aerobic capacity, anaerobic power, total work, fatigue index, and RPE	Before and after the testing protocol	Lower body compression garments during activity	↑* mean power output<br>↑* anaerobic capacity<br>↑* total work<br>↓* RPE<br>↔ peak power output<br>↔ anaerobic power<br>↔ fatigue index
Chaiyakul & Chaibal (2021)	11 university male basketball players	HIIE (4x20m shuttle sprint and repetitive vertical jumps)	20m shuttle sprint, VJ_{max} HR, and RPE	Baseline and 24h after HIIE protocol	Cold water immersion (15℃) 1h and 3h after completion of exercise	↑* VJ_{max} (1h and 3h)<br>↓* muscle soreness (1h and 3h) vs. control

Study	Subjects	Exercise protocol	Measurement	Measurement timing	Recovery methods	Findings
Cordova Martinez et al. (2017)	12 professional male basketball players	2h gym workout in the morning and 3h basketball practice in the afternoon	CK, Mb, AST, ALT, LDH, urea, creatinine, TP, testosterone, cortisol, ACTH	Baseline, Day 1 and, and Day 20	6g/day (20 days) of L-glutamine supplementation	↓* CK, ACTH<br>↓* Mb, AST
Delextrat et al. (2013)	8 male and 8 female university basketball players	Competitive match	CMJ, RSA, perception of fatigue	24h after competitive match	Massage<br>CWI	↓* Perception of fatigue (massage and CWI)<br>↑* CMJ (CWI)
Delextrat et al. (2014)	9 male and 8 female university basketball players	Competitive match	Perception of fatigue, leg soreness, CMJ, and RSA	Perception of fatigue and leg soreness immediately and 24h post-treatment and CMJ and RSA 24h post-treatment	Massage<br>MAS	Men: ↑* CMJ for both treatments<br>Women: MAS ↑* RSA<br>Women > Men
Fernández-Lázaro et al. (2020)	12 professional (treatment group) and 12 university basketball players (control group)	2x/day training sessions for 5 days in a row with 1 day of rest, staggered with 6 friendly matches	RPE (Borg 10-scale), LEU, MON, LIN, RBC, Hb, Hct, Mb, CK, LDH, AST, and ALT	Baseline (T1), Week 4 (T2), and Week 8 (T3)	After each training session and match players received compressive cryotherapy (2x15 min at 8°C with 5 min rest)	↓* Hb in treatment group (T2 and T3 compared to T1)<br>↓* RPE in treatment group (T2 and T3 compared to T1)<br>↑* CK activity in the control group (T3 compared to T1)<br>↓* Mb in the treatment group (T3 and T2 compared to T1)
Ho et al. (2018)	15 collegiate male basketball players	1h cycling at 70% VO_{2max}	Cerebral oxygen saturation, glucose, insulin, and cycling time	Baseline and after the exercise protocol completion	High-protein ingestion (6.25 kcal/kg; 6% fat, 36% protein, 58% carbohydrate)	↑* Cerebral oxygen saturation<br>↑* Peak insulin level<br>↓* Glucose level<br>↑* Longer cycling time
Hoffman et al. (2012)	10 female NCAA Division-I athletes	4x40 min basketball game with 10-min half time	CMJ, 20s reaction time, player load, and basketball shooting assessment	Pre-post game	L-alanyl-L-glutamine ingestion (AG1-1g/0.5L), (AG2-2g/0.5L), or water	↑* shooting performance (AG1 vs. non-hydrated)<br>↑* shooting performance (AG1 vs. water)<br>↑* player load (AG2 vs. non-hydrated)

Study	Subjects	Exercise protocol	Measurement	Measurement timing	Recovery methods	Findings
Howard & Hillman (2016)	8 female NCAA Division-III basketball players	Single NCAA half game regulation	CVJ, T-test, lay-ups made, jump shots made, 300-yard shuttle run, memory test, reaction time, plasma volume, blood lactate	Pre-post exercise protocol	Hydration interventions with carbohydrate-electrolyte solution (CES) vs. water (placebo)	↓* plasma volume by 1.3±4.0% in CES vs. 3.2±5.0% in placebo<br>↑* memory test when consuming CES<br>↓* plasma volume change when consuming CES<br>↓* blood lactate with increase in CES ingestion
Kaesaman & Eungpinichpong (2019)	16 amateur male basketball players	20 min basketball simulated game	HRV and physical fitness (sit-and-reach test, grip strength, leg strength, and back strength)	Baseline, pre-recovery and post-recovery	10 min traditional Thai massage immediately post-exercise	↑* HRV
Lin et al. (2017)	11 male NCAA Division-II basketball players	4x10 min quarters with 10 basketball drills and two 60 s rest in each quarter	Basketball skill test, RPE, CK, LDH, T, C, T/C, lactate, glucose, and glycerol	Before exercise, post-first half and post-second half on Day1 and Day2	0.17g/kg BCAA (2:1:1) and 0.04g/kg arginine	↓* total time to complete vertical jumps and ladder suicide sprint post-second half on Day2 in the intervention group<br>↓* post-exercise free tryptophan/BCAA in the intervention group<br>↔ shooting, dribbling, and passing skills
Lin et al. (2009)	30 university male basketball players	Cycling until complete exhaustion (60 RPM at 120W, with 30W increase every 2 min)	HR, HR_{max}, VO_{2max}, and blood lactate	Rest, 5 min, 30 min, and 60 min post-exercise	Acupuncture at the Neiguan (PC6) and Zusanli (ST36) acupoints	↓* HR_{max}<br>↓* VO_{2max}<br>↓* blood lactate concentration
Mah et al. (2011)	11 male NCAA Division-I basketball players	N/A	Sprint time, shooting accuracy, POMS, ESS, PVT, and sleep-wake activity	After every practice on weekly bases, before and after the period of extended sleep	Extended sleep (>10h)	↑* sleep time<br>↑* faster sprint times<br>↑* free-throw and three-point percentage<br>↓* mean PVT reaction time and ESS scores<br>↑* POMS with increased vigor and decreased fatigue<br>↑ ratings of physical and mental well-being

Study	Subjects	Exercise protocol	Measurement	Measurement timing	Recovery methods	Findings
Montgomery et al. (2008a)	29 amateur male basketball players	3-day basketball tournament	Fatty-acid binding protein (FABP), CK, Mb, IL-6, IL-10, and muscle soreness	Baseline, 10min, 6h and 24h post-game	CHO and stretching, CWI, and full-leg compression garments	↓ post-tournament rise in FABP and Mb<br>Moderate change in CK<br>↓ post-game muscle soreness after CWI
Montgomery et al. (2008b)	29 amateur male basketball players	3-day basketball tournament	Physical performance tests (sprint, agility, CVJ, 20m acceleration and time, sit-and-reach), and muscle soreness	Before and after 3-day basketball tournament	CHO and stretching, CWI, and full-leg compression garments	↑* performance tests for CWI than CHO and stretching and full-leg compression garments<br>↓ CWI muscle soreness
Pelana et al. (2020)	26 university male basketball players	High-intensity interval training	Blood lactate and CVJ	Before and after the recovery session	CWI 16ºC for 7 min or slow jogging for 8 min	↓* blood lactate post-recovery for CWI vs. slow jogging
Ronghui (2015)	10 university male basketball players	Ergometer cycling with progression of the load every 5 min (20 min total)	Hb, RBC, HCT, and MCV	Before and after one month trial	20g whey protein and 40g oligosaccharides dissolved in 250ml milk	↑* Hb, RBC, HCT in intervention vs. control group
Schröder et al. (2000)	24 professional male basketball players	32-day basketball competitive season	Blood samples (LPO, TAS, Vitamin C, retinol, α-tocopherol, vitamin C, β-carotene)	Before and after supplementation	4x/day ingestion of 150mg α-tocopherol, 250mg ascorbic acid, and 8mg β-carotene	↑* plasma α-tocopherol and β-carotene in the experimental group<br>↓ plasma lipoperoxide, retinol, and vitamin C post-treatment with the antioxidants<br>↓ LPO/TAS ratio
Seco-Calvo et al. (2020)	28 professional basketball players	Full basketball competitive season	RPE, isokinetic peak torque, and muscle metabolism serum markers (lactate, Mb, CK, aldolase, glutamate oxalacetate transaminase, urea, creatinine, aspartate aminotransferase)	RPE and isokinetic peak torque (pre and post-season), blood samples (4x/season and 24h post last game)	CWI (10.5°C) - 5x2min intermittent whole-body immersions, separated by 2 min rest in ambient air (20.7°C)	↓* RPE for CWI from the beginning to the end of the season<br>↑* isokinetic strength (CWI vs. control group)<br>↑ serum markers at each testing timepoint during the season were higher for CWI, except Mb

Study	Subjects	Exercise protocol	Measurement	Measurement timing	Recovery methods	Findings
Svilar et al. (2019)	13 professional male basketball players	3-8 training sessions during the season and 2-3 games/week (10 games total)	External load metrics (player load, number of jumps, accelerations, and decelerations, changes of direction - total and high-intensity thresholds), RPE, and TQR	RPE 15-30-min following the training session, external load metrics during the training sessions, and TQR on game day and after morning practice	Short-term tapering (3 days pre-game)	↑ TQR on a match day
Vidic et al. (2017)	13 female NCAA Division-I basketball players	Competitive basketball season (November-March)	Perception of Stress Scale-10, Athletic Skills Coping Inventory-28, Qualitative Reflections	Pre-season, mid-season, and post-season	10-20 minutes of education regarding mindfulness and 15-20 minutes of guided meditation	↓* perceived stress (pre to mid-test and mid to post-test)<br>↑* athletic coping skills (pre to mid and post-test and mid to post-test)
Watson et al. (2020)	19 male NCAA Division-I basketball players	2 consecutive basketball seasons	RPE, well-being variable (mood, fatigue, soreness, stress, sleep duration), and injury incidence	Every day during the competitive season	Sleep duration	↓* injury incidence with increased sleep duration (>8h of sleep)
Zhao et al. (2012)	20 amateur female basketball players	14 days of identical training sessions	Sleep quality, Cooper 12 min run (endurance), serum melatonin	Before and after intervention protocol	30 min red-light therapy	↑* sleep quality<br>↑* serum melatonin and endurance performance

↑ = increased; ↑* = significantly increased; ↓ = decreased; ↓* = significantly decreased; ↔ = no changes; AST = aspartate aminotransferase; ACTH = adrenocorticotropic hormone; ALT = alanine transaminase; BCAA = branched chain amino acids; C = cortisol, CHO = carbohydrates; CK = creatine kinase; CMJ = countermovement vertical jump; CWI = cold-water immersion; ESS = Epworth Sleepiness Scale; Hb = hemoglobin; Hct = hematocrit; HIIE = high-intensity interval exercise; HR = heart rate; HR_{max} = maximal heart rate; HRV = heart rate variability; IL-6 = interleukin-6; IL-10 = interleukin-10; LEU = leucocytes; LIN = lymphocytes; LDH = lactate-dehydrogenase; LPO = lipoperoxides; LPO/TAS = lipoperoxides; TO = total antioxidant status ratio; MAS = massage and stretching group; Mb = myoglobin; MON = monocytes; MVIC = maximal voluntary isometric contraction; $NaHCO_3$ = sodium-bicarbonate; NCAA – National Collegiate Athletic Association; POMS = profile of mood states; PVT = Psychomotor Vigilance Task; RBC = red blood cells; RPE = rating of perceived exertion; RST = repeated sprint test; RSA = repeated sprint ability; TAS = total antioxidant status; T = testosterone; T/C = testosterone-to-cortisol ratio; TQR = Total Quality of Recovery; VJ_{max} = maximal vertical jump height; VO_{2max} = maximal oxygen consumption.
